# Is LEA symbol better compared to Snellen chart for visual acuity assessment in preschool children?

**Published:** 2019

**Authors:** Undrakonda Vivekanand, Sarita Gonsalves, Shailaja S Bhat

**Affiliations:** * Department of Ophthalmology, Alluri Sitaramaraju Academy of Medical Sciences, Andhra Pradesh, India; ** Department of Ophthalmology, Father Muller’s Medical College, Mangalore, Karnataka, India; *** Department of Ophthalmology, Kasturba Medical College, Manipal, Karnataka, India

**Keywords:** LEA symbols, preschool children, Snellen chart, visual acuity

## Abstract

**Aim:** To compare visual acuity using the LEA symbol chart with Snellen E test chart in preschool children of age 3-5 years.

**Patients and methods**:

**Inclusion criteria:** 50 emmetropic children aged 3 to 5 years.

**Exclusion criteria:** Strabismus, amblyopia, ametropia, and any organic eye disease. A pseudo randomized protocol was used to test visual acuity (VA) in each subject monocularly on both eyes using Snellen E chart and LEA symbol chart. Visual acuity for both charts was scored as smallest optotype size which the child correctly identified 3 of maximum 4 optotypes. The strength of agreement on VA between two charts was tested using Interclass correlation coefficient (ICC). A Mann-Whitney U test was applied to compare both the groups.

**Results: **Boys: Girls = 26:24 with a mean age and standard deviation of 4.12 + 0.79 years. ICC between Snellen’s and LEA symbol chart was 0.256 and 0.213 for right and left eye respectively. Analysis of the two samples using Mann-Whitney test showed a significant difference between the two charts (p value <0.000).

**Conclusion: ** LEA symbol test showed only a fair agreement with Snellen E charts for visual acuity measurements. Visual acuity measurement with LEA symbol chart showed significantly higher scores as compared to Snellen’s chart.

## Introduction

An ideal visual acuity screening test in paediatric age group needs to be a simple, accurate, and reproducible method. LEA symbol charts are designed to eliminate problems associated with language barriers. The symbols are easy to recognize and accessories are available to create a “play situation”, making screening easier and more accurate. Literature review reveals 23 months as the earliest age at which LEA symbol could be used to visual acuity in a child. Hence, LEA symbol can be used to assess visual acuity in children older than 30 months of age [**1**]. The present study compared visual acuity results obtained using the LEA symbol chart with that of Snellen E test chart in children without any eye or neurological problems of age group 36 months to 60 months.

## Patients and methods

50 emmetropic children between ages 3 to 5 years (preschool) were recruited in this study. Each child had a basic orthoptic assessment, which included cover test for distance and near, ocular motility, retinoscopy, fundus red reflex test, Hirschberg test [**2**]. Children with strabismus, amblyopia, ametropia, and any other organic eye disease were excluded from the study. After obtaining an informed consent from parents, all the children underwent visual acuity assessment monocularly on both eyes using Snellen E chart (at 6m) and LEA symbol chart (at 3 m). The order of the visual acuity tests was performed using a pseudo-randomized protocol so that there was an equal chance to start testing either with LEA symbols or with the Snellen E chart. Letter matching with an appropriate key card or a verbal response (2 of 3 correct responses) was taken for assessment. The examiner conducting the test was blinded and had no knowledge of any results of previous eye tests. Scoring of visual acuity on LEA symbol done after child identified 3 out of maximum 4 smallest optotypes correctly [**3**]. Scoring for Snellen’s chart was performed after child identified at least 3 letters correctly. Single optotype acuity was converted to modified logMAR to allow a direct examination of the two scoring systems. 

The strength of agreement between the two visual acuity charts was evaluated using the Interclass correlation coefficient. A Mann-Whitney U test was applied to compare both the groups. Statistical data analysis was performed using Windows Microsoft excel software. 

## Results

Of the 50 children tested, there were 26 (52%) boys and 24 (48%) girls with an overall mean age and standard deviation (SD) of 4.12 + 0.79 years. ICC between Snellen’s and LEA symbol chart was 0.256 and 0.213 for the right and left eye respectively (**Table 1**). 

**Table 1:** Interclass coefficient between Snellen’s and LEA symbol chart

Chart	Visual acuity	Interclass correlation coefficient	
		Right eye	Left eye
LEA	3/ 3 -3/ 6		
Snellen E	6/ 6 -6/ 12	0.213 {CI (95%) = -0.08 to 0.47}	0.256{CI(95%) = -0.41 to 0.51}

The mean LogMAR visual acuity in the right eye was 0.196 and 0.074 using Snellen’s chart and LEA symbol chart respectively. The mean LogMAR visual acuity in the Left eye was 0.144 and 0.064 using Snellen’s chart and LEA symbol chart respectively. The P values using Mann Whitney U test were statistically significant showing visual acuity measurement with LEA symbol chart being better than Snellen’s chart (**Table 2**). 

**Table 2:** P Values and LogMAR visual acuity values of right eye and left eye

	Right eye		Left eye	
	*Snellen’s Chart*	*LEA Chart *	*Snellen’s Chart*	*LEA Chart *
Mean (logMAR)	0.196	0.074	0.144	0.064
P value	0.000000000140		0.000302055	

## Discussion

Picture based charts play a significant role in quantitatively evaluating visual acuity in preschool children. LEA Symbols chart is designed to eliminate problems associated with language barriers. Dr Lea Hyvärinen designed a set of tests based on picture optotypes for use in children. These tests make use of common pictures believed to improve test ability among young children and eliminate cultural biases. The chart gives high sensitivity for measuring visual acuity in both childhood (age 4 and above) with early and reliable detection of amblyopia. Previous studies have found that in older children, visual acuity assessment using LEA symbols’ is 0.5 to 2 lines better than non-logMAR Landolt C charts or with Bailey-Lovie logMAR letter charts. In addition, preliminary results from 1st grade children found LEA symbols visual acuity to be approximately 0.5 lines better than ETDRS visual acuity [**4**,**5**]. In our study, we found that LEA symbols optotype sizes visual acuity scores showed a fair agreement and better quantitative assessment of visual acuity level as compared to Snellen E chart.

## Conclusion

LEA symbols test are better for visual acuity assessment as compared with Snellen E charts for visual acuity measurements in preschool children.

**Financial support and sponsorship**

Nil.

**Conflict of interest**

The authors declare that there is no conflict of interests.

This paper was presented as a poster in 74th Conference of All India Ophthalmic Society held between the 25th to 28th February 2016, Kolkata, India.

## References

[1] Becker RH, Hübsch SH, Graf MH, Kaufmann H (2000). Preliminary report: examination of young children with Lea symbols. Strabismus.

[2] Amitava AK, Kewlani D, Khan Z, Razzak A (2011). Assessment of a modification of Bruckner's test as a screening modality for anisometropia and strabismus. Oman Journal of Ophthalmol.

[3] Hyvärinen L (2017). LEA vision test system for assessment and screening. [Online] Good lite.

[4] Lithander J (1996). Two techniques to evaluate visual acuity from the age of 18 months. Strabismus.

[5] Dobson V, Clifford-Donaldson CE, Miller JM, Garvey KA, Harvey EM (2009). A comparison of Lea Symbol vs. ETDRS letter distance visual acuity in a population of young children with a high prevalence of astigmatism. J AAPOS.

